# P-320. Beyond race: Predicting HIV acquisition through sociostructural and healthcare utilization factors in Atlanta

**DOI:** 10.1093/ofid/ofaf695.539

**Published:** 2026-01-11

**Authors:** Meredith H Lora, Megan Schwinne, Chad Robichaux, Andres Camacho-Gonzalez, Reza Sameni, Amelia Muniz Hernandez, Rishika Iytha, Siri Chirumamilla, Emma J Hollenberg, Sarah Gruber, Valeria D D Cantos

**Affiliations:** Emory University School of Medicine, Atlanta, GA; Emory University, Atlanta, Georgia; Emory University, Atlanta, Georgia; Emory University School of Medicine, Atlanta, GA; Emory University, Atlanta, Georgia; Private Practice, Rego Park, New York; Georgia Institute of Technology, Pittsburgh, Pennsylvania; Emory University School of Medicine, Atlanta, GA; Hospital of the University of Pennsylvania, Philadelphia, Pennsylvania; Emory University School of Medicine, Atlanta, GA; Emory University School of Medicine, Atlanta, GA

## Abstract

**Background:**

HIV disproportionately affects Black individuals, yet HIV pre-exposure prophylaxis (PrEP) is underutilized in this group. Racial disparities stem from sociostructural factors and low healthcare access rather than behavioral risk. Machine learning models identifying persons at risk for HIV may increase PrEP uptake. Previous HIV prediction models have included race as a significant predictor, which may perpetuate stigma towards the Black population.

**Figure:**
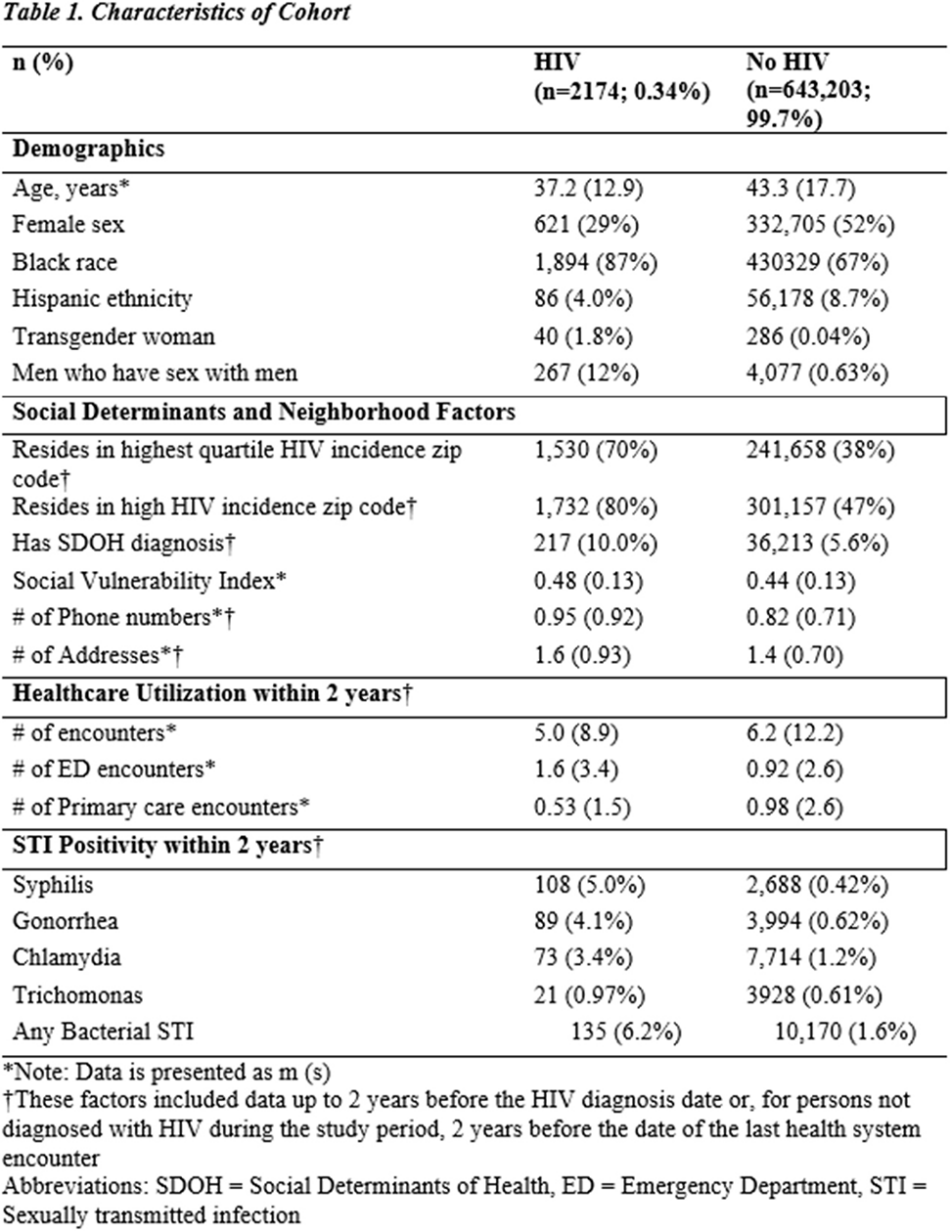

**Figure:**
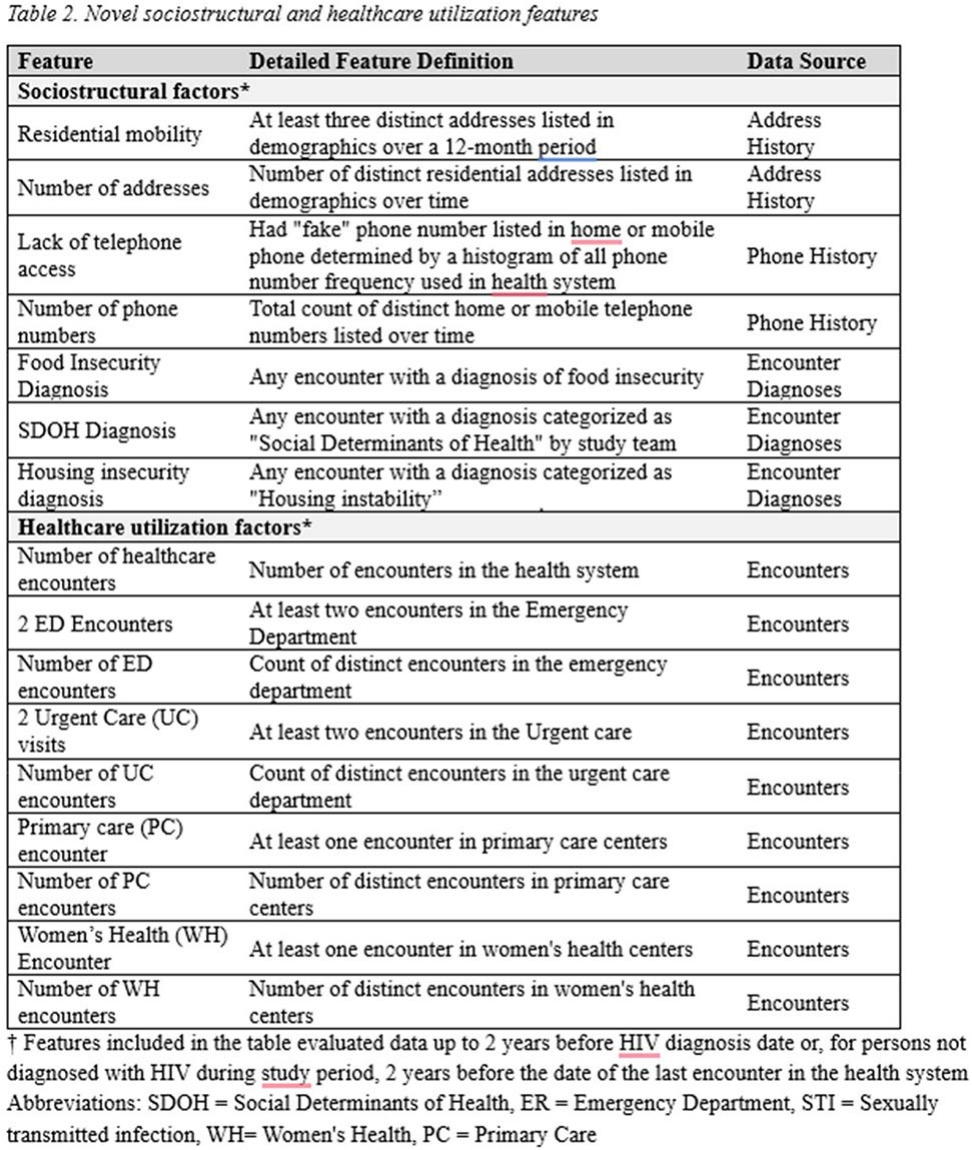

**Methods:**

We developed an electronic health record (EHR)-based machine learning model to predict HIV diagnosis among adult patients seen at the Grady Health System in Atlanta from 2012 to 2022. We developed over 160 potential predictors, including 16 novel sociostructural and healthcare utilization features. We used an XGBoost classifier to train the model (80-20 train-test split) and quantified feature importance using Shapley Additive Explanations (SHAP). We then removed race and ethnicity features and retrained the model using the same approach. We evaluated the model using area under the receiver operating characteristic and precision-recall curves (AUROC, AUPRC).

**Figure 1.**
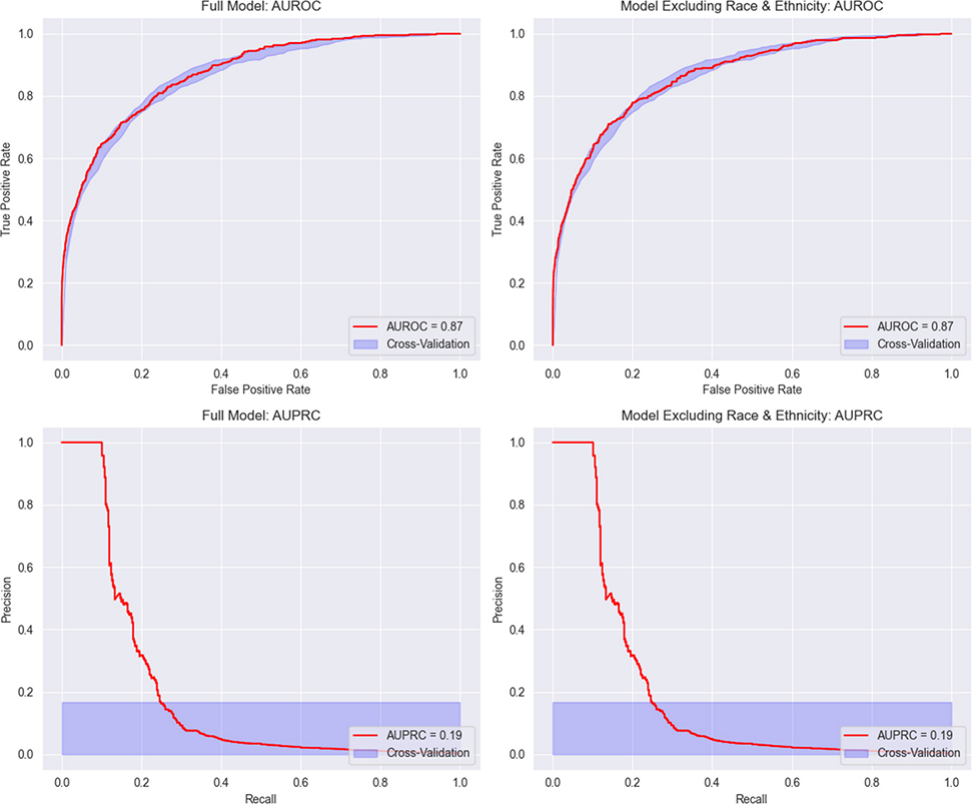
Model performance

Model performance by AUROC and AUPRC. AUROC indicates the model's ability to discriminate between true positives and negatives, with a baseline of 0.5 for random guessing. AUPRC is used in imbalanced datasets to measure the model's ability to distinguish true positives from false positives, with a baseline equal to the outcome incidence (0.0034 in this population). A higher AUPRC than the baseline suggests effective identification of true positives. A: AUROC—model including race and ethnicity features. B: AUROC—model excluding race and ethnicity features. C: AUPRC—model including race and ethnicity features. D: AUPRC—model excluding race and ethnicity features. Abbreviations: AUROC, area under the receiver operating characteristic curve; AUPRC, area under the precision-recall curve.

**Figure 2.**
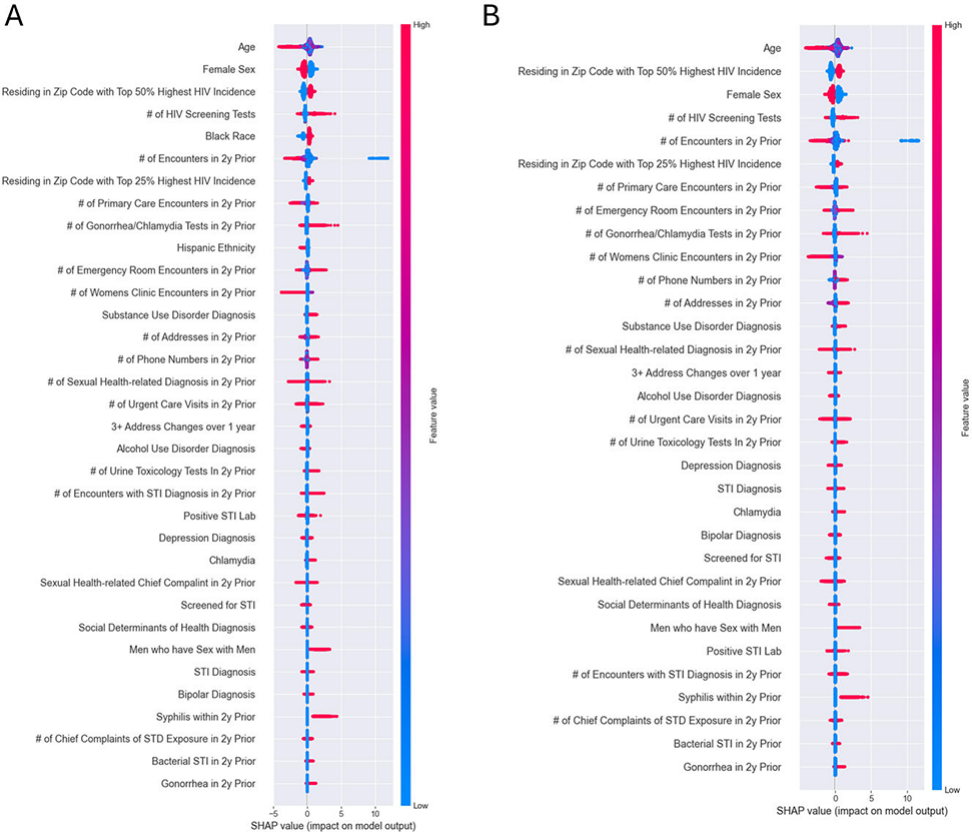
Shapley additive exPlanations from XGBoost Model

The SHAP (SHapley Additive exPlanations) values show the importance of each feature in the model. These values help explain how each feature contributes to the model's predictions, using principles from cooperative game theory to approximate the complex model with a simpler linear explanation." A. Model including race and ethnicity B, Model excluding race and ethnicity. Abbreviations: SHAP = SHapley Additive exPlanations, 2y = 2 years, SDOH = Social Determinants of Health, STI = Sexually transmitted infection, STD = Sexually transmitted disease, # = Number. Features evaluating data "2y prior" included data up to 2 years before the HIV diagnosis date or, for persons not diagnosed with HIV during the study period, 2 years before the date of the last health system encounter.

**Results:**

Of 645,377 individuals, 2,174 (0.34%) received an HIV diagnosis; 87% were Black, 29% women, and 70% lived in high-incidence areas. The race-agnostic model had an AUROC of 0.87 and an AUPRC of 0.19, matching the race-inclusive model. Key features included sociostructural factors (residency in high HIV incidence areas and frequent changes to phone number and address) and healthcare utilization patterns (seeking HIV and STI testing, more emergency and fewer primary care visits). Other key features were younger age and male sex. Positive gonorrhea or syphilis and identifying as a man who has sex with men were less predictive than sociostructural and healthcare utilization factors.

**Conclusion:**

Our first in-class, race-agnostic HIV prediction model achieved comparable predictive accuracy to a race-inclusive model in a predominantly Black population in a high HIV incidence area. Sociostructural and healthcare utilization features influenced the model more than behavioral or STI diagnosis factors. Our model may help dispel misconceptions of race as a biological or behavioral risk factor for HIV and merits further study.

**Disclosures:**

Meredith H. Lora, MD, Viiv healthcare: Grant/Research Support Andres Camacho-Gonzalez, MD, MSc, Gilead Sciences Inc.: Grant/Research Support|GSK: Grant/Research Support|Merck: Grant/Research Support|ViiV: Grant/Research Support Valeria D. D. Cantos, MD, FIDSA, Gilead Sciences Inc.: Grant/Research Support

